# Diagnostic utility of targeted flow cytometry in genetically confirmed inborn errors of immunity: a 20-year single-center experience

**DOI:** 10.3389/fimmu.2026.1940152

**Published:** 2026-09-24

**Authors:** Özgen Soyöz, Figen Çelebi Çelik, Mehmet Şirin Kaya, Ayça Demir, İlke Taşkırdı, İdil Akay Hacı, Selime Özen Bölük, Murat Sürücü, Nesrin Gülez, Ferah Genel

**Affiliations:** 1 İzmir Bakırçay University Çiğli Training and Research Hospital, Department of Pediatric Allergy and Immunology, Izmir, Türkiye; 2 University of Health Sciences, İzmir Dr. Behçet Uz Child Disease and Pediatric Surgery Hospital, Department of Pediatric Allergy and Immunology, İzmir, Türkiye; 3 İzmir Katip Çelebi University, Department of Pediatric Allergy and Immunology, , İzmir, Türkiye; 4 Adnan Menderes University, Department of Pediatric Allergy and Immunology, Aydın, Türkiye

**Keywords:** chronic granolumatous disease, combined immunodeficiencies, flow cytometry, human inborn errors of immunity, next-generation sequencing

## Abstract

**Background:**

Inborn errors of immunity (IEIs) are a heterogeneous group of genetic disorders in which timely diagnosis is essential for appropriate treatment and improved clinical outcomes. Although next-generation sequencing provides definitive molecular confirmation, it is often time-consuming, costly, and may not identify all disease-causing variants. We evaluated the diagnostic performance and diagnostic contribution of targeted flow cytometry (FCM) in genetically confirmed IEIs.

**Methods:**

We retrospectively analyzed 243 pediatric patients with genetically confirmed IEIs who underwent targeted flow cytometric evaluation at a single center between 2005 and 2025. Immunological evaluation included T-, B-, and NK-cell immunophenotyping, detailed T- and B-cell subset analysis, targeted protein expression assays, and neutrophil oxidative burst assessment using the dihydrorhodamine 123 assay. Molecular diagnoses were established by targeted gene panels, whole-exome sequencing, or Sanger sequencing.

**Results:**

The median age at symptom onset and molecular diagnosis was 12 and 70 months, respectively. Predominantly antibody deficiencies (31.7%) and syndromic combined immunodeficiencies (27.1%) were the most frequent IEI categories. Abnormal FCM findings were detected in 153 patients (63%) and predicted the underlying genetic category in 41 (27%). Diagnostic yield reached 100% in combined immunodeficiencies and 82.6% in immune dysregulation disorders, significantly exceeding that of syndromic combined immunodeficiencies and antibody deficiencies (*p* < 0.05). Importantly, FCM findings preceded molecular confirmation by a median of 12 months.

**Conclusions:**

Targeted flow cytometry provides rapid functional evidence that complements molecular testing in patients with IEIs. Its integration into the initial diagnostic evaluation facilitates early disease stratification, guides targeted genetic investigations, and supports timely therapeutic decision-making.

## 1 Introduction

Human inborn errors of immunity (IEIs) are a broad, heterogeneous group of diseases resulting from genetic defects that clinically lead to recurrent, chronic bacterial, viral, and fungal infections, as well as an increased susceptibility to autoimmunity and malignancy (1, 2). Although it is known as a rare disease, a recent study in the USA reported a prevalence of IEIs of 6 per 10,000 people, indicating that they are less rare than previously assumed (3). To date, hundreds of different genetic defects have been identified as causing IEIs. Since the advent of next-generation sequencing technologies, the number of identified disorders—including both rare and common genetic defects—has increased markedly (4). These can be autosomal or X-linked, inherited recessively or dominantly, and present with incomplete or complete penetrance of the clinical phenotype. A total of 559 IEIs were identified in the 2025 update of the phenotypic classification of the International Union of Immunological Societies (IUIS) expert committee on IEIs (1).

Although IEIs can be diagnosed based on clinical findings and hematological/biochemical laboratory tests according to European Society for Immunodeficiencies (ESID) diagnostic criteria (5), accurate diagnosis may not always be possible due to the heterogeneity of clinical phenotypes. However, early and accurate diagnosis is crucial for initiating appropriate life-saving treatments and thus reducing associated morbidity and mortality. Genetic analyses play a significant role in accurate diagnosis, but these analyses are time-consuming, costly, and laborious. Access to genetic testing is not readily available in every country. Furthermore, targeted gene tests may miss some mutations. Exome sequencing, which has been used in the diagnosis of many genetic diseases in recent years, can only detect causative genetic defects in 40% to 58% of patients (6, 7).

Over the past few decades, flow cytometry has evolved into a fundamental tool for the evaluation of the immune system. It enables both the quantitative assessment and detailed phenotypic characterization of immune cell populations, thereby supporting the diagnosis of a wide range of IEIs. With its increasing accessibility, this technique is now routinely employed in clinical practice for diagnostic purposes (8). In addition to immunophenotyping, it allows real-time functional analysis of immune cells, facilitating the detection of cellular defects associated with IEIs. Consequently, it serves as a valuable first-line screening approach in patients with suspected immunodeficiency (9). Given that many IEIs arise from abnormalities in the number, distribution, or function of immune cells and their associated proteins, flow cytometry is integral to the diagnosis and classification of these disorders, as parameters such as absolute T-cell counts, total B-cell numbers, and class-switched memory B-cell subsets contribute to established diagnostic criteria for conditions including severe combined immunodeficiency, X-linked agammaglobulinemia, common variable immunodeficiency and chronic granulomatous disease (1, 5, 8–11).

In this study, we retrospectively evaluated targeted flow cytometric analyses performed over a 20-year period in a cohort of 243 patients with genetically confirmed IEIs. We aimed to assess the diagnostic contribution and clinical utility of targeted flow cytometry by comparing flow cytometric findings with the final molecular diagnoses across different IEI categories.

## 2 Materials and methods

### 2.1 Patients and data collection

Patients aged 0–18 years who were evaluated at the Pediatric Immunology and Allergy Clinic of University of Health Sciences İzmir Dr. Behçet Uz Children’s Diseases and Surgery Training and Research Hospital between 2005 and 2025, fulfilled the European Society for Immunodeficiencies (ESID) clinical diagnostic criteria for IEIs, and had a genetically confirmed diagnosis were included in this retrospective study. Patients were subsequently classified according to the 2025 update of the International Union of Immunological Societies (IUIS) classification of inborn errors of immunity. Patients with autoinflammatory diseases or inherited bone marrow failure syndromes were excluded.

Demographic and clinical data, including age, sex, presenting manifestations, age at symptom onset, age at diagnosis, family history, molecular diagnosis, and flow cytometric findings, were retrospectively retrieved from electronic medical records and the hospital database.

The study was approved by the Ethics Committee of the University of Health Sciences, İzmir Dr.Behçet Uz Children’s Diseases and Surgery Training and Research Hospital (approval No. 13399118-799; September 22, 2020).

### 2.2 Flow cytometry evaluation

Peripheral blood immunophenotyping was performed in all patients as part of the initial immunological evaluation. Baseline lymphocyte subsets were analyzed by flow cytometry using lineage-specific markers, including CD3 for total T cells, CD4 and CD8 for helper and cytotoxic T-cell subsets, CD19 for B cells, and CD16/CD56 for natural killer cells. Lymphocyte populations were identified according to their forward- and side-scatter characteristics in combination with CD45 expression.

Standard laboratory investigations, including complete blood count with differential, peripheral blood smear evaluation, serum immunoglobulin levels, and complement measurements, were performed in all patients. Based on the clinical presentation, routine laboratory findings, and initial immunophenotypic results, additional targeted multiparametric flow cytometric analyses were performed when indicated. Selection of these assays was guided by clinical features, including age at disease onset, infection pattern, and the presence of immune dysregulation, autoimmunity, or inflammatory manifestations. Specialized flow cytometric assessment of complement-related cell-surface protein expression was not routinely available during the study period and was not included in the evaluated panel. The complete panel of flow cytometric analyses performed in this study is summarized in **Table 1**.

**Table 1:** Immunological evaluation of inborn errors of immunity: lymphocyte subset characterization, assessment of disorder-related protein expression, and immune cell functional testing.

Test	Cell populations	Indications
Phenotypic characterization of peripheral blood lymphocyte populations		
T-B-NK phenotyping	CD3 +T cells, CD4+ T cells, CD8+ T cells, CD19+B cells, NK cells	Basic screening for IEI, SCID, CID, PAD
T-cell phenotyping		
T1 panel	CD4+ Naive (CD4+CD45RA+), memory (CD4+CD45RO+), CD8+ Naive (CD8+CD45RA+), CD8+ memory (CD8+CD45RO+), Central memory CD4+ (CD4+CD45RA-CD27+), Naive CD4+ (CD4+CD45RA+CD27+), Effector memory CD4+ (CD4+CD45RA -CD27-), Terminal effector memory CD4+ (CD4+CD45RA+CD27-), Central memory CD8+ (CCR7+CD8+CD45RA -CD27+), Naive CD8+ (CCR7+CD8+CD45RA+CD27+), Effector memory CD8+ (CCR7-CD8+CD45RA -CD27+) Terminal Effector memory CD8+ (CD8+CD45RA +CD27-)	SCID, leaky SCID, OS, CID, CVID
T2 panel	RTE (CD4+CD45RA+CD31+)	SCID, OS, leaky SCID, DiGeorge syndrome, CID
T4 panel	TCRαβ DNT (CD3^+^TCRαβ^+^CD4^−^CD8^−^)	ALPS
T5 panel	Treg (FoxP3^+^CD25^+^)	IPEX syndrome
B-cell phenotyping		
B panel	Switched memory (CD27+sIgD−), non-switched memory (CD27+sIgD+), transitional B cells (CD38hiCD24hi), plasmablasts (CD38hiCD24−) and CD21low B cells (CD38lowCD21low)	CVID, HIMS, PAD, CID, LRBA deficiency, RIPK1 deficiency
Analysis of the expression of specific surface or intracellular proteins		
HLA-DR	B cells	CID with low CD4 (MHC-II deficiency)
CD40L	PMA/Ionomycin stimulated CD4+ T cells	X-linked HIMS
CD40	B cells	HIMS
CD18/CD11a	Neutrophils	LAD-1
CD15	Neutrophils	LAD-2
Functional assays		
Oxidative burst assay (DHR 123 assay)	Neutrophils	CGD, EO-IBD

ALPS, autoimmune lymphoproliferative syndrome; CGD, chronic granulomatous disease; CID, combined immunodeficiency; CVID, common variable immunodeficiency; DHR, Dihydrorhodamine; EO-IBD, early onset inflammatory bowel disease; HIMS, hyper-IgM syndrome; HLA, human leukocyte antigen; IEI, inborn errors of immunity; IPEX, immune dysregulation-polyendocrinopathy-enteropathy-x-linked; LRBA, LPS-responsive beige-like anchor protein; LAD, leukocyte adhesion deficiency; MHC, major histocompatibility complex; OS, Omenn syndrome, PAD, predominantly antibody deficiency; PMA, phorbol-12-myristate-13-acetate; RIPK1, receptor-interacting serine/threonine-protein kinase 1; RTE, recent thymic emigrants; SCID, severe combined immunodeficiency; sIgD, surface IgD; Treg, regulatory T cells.

#### 2.2.1 T-cell phenotyping

Comprehensive T-cell immunophenotyping was performed in patients with T-cell lymphopenia or clinical and laboratory findings suggestive of Omenn syndrome, atypical severe combined immunodeficiency, or combined immunodeficiency. Naïve and memory T-cell compartments were evaluated using multicolor flow cytometry panels incorporating CD3, CD4, CD8, CD45RA, CD45RO, CD27, and CCR7, enabling the identification of naïve, memory, and terminally differentiated effector subsets within both CD4+ and CD8+ T-cell populations (**Table 1**).

In selected patients, recent thymic emigrants were identified by assessing CD31 expression in combination with CD45RA on CD4+ T cells. Additional targeted flow cytometric assays were performed according to the suspected diagnosis. In patients with suspected autoimmune lymphoproliferative syndrome, TCRαβ double-negative T cells were quantified using panels designed to distinguish αβ and γδ T-cell receptor expression in conjunction with conventional T-cell markers. Regulatory T-cell populations were assessed by intracellular FOXP3 staining or by established surface marker combinations, particularly in patients with clinical features suggestive of immune dysregulation syndromes such as immune dysregulation, polyendocrinopathy, enteropathy, X-linked (IPEX) syndrome.

#### 2.2.2 B-cell phenotyping

Detailed characterization of peripheral B-cell subsets was performed in patients with suspected predominantly antibody deficiencies, particularly those with preserved or only mildly reduced circulating B-cell counts. Multiparametric flow cytometry panels incorporating CD19, CD27, surface IgD, CD24, CD38, and CD21 were used to define distinct B-cell populations. These analyses enabled the identification of naïve, transitional, non-class-switched memory, class-switched memory, plasmablast, and CD21^low^ B-cell subsets based on their characteristic immunophenotypic profiles (**Table 1**).

#### 2.2.3 Assessment of cell surface and intracellular protein expression by flow cytometry

##### 2.2.3.1 Cell surface protein expression

Targeted flow cytometric analysis was performed to assess the expression of selected cell surface and intracellular proteins according to the suspected underlying IEI (**Table 1**). Both the proportion of positive cells and fluorescence intensity values were quantified and interpreted in comparison with reference controls.

Patients with clinical and immunological features suggestive of major histocompatibility complex (MHC) class II deficiency were assessed for HLA-DR expression on B lymphocytes and monocytes. In patients with suspected hyper-IgM syndrome, CD40 expression on B cells and inducible CD40 ligand (CD154) expression on activated CD4+ T cells were evaluated following *in vitro* stimulation. In addition, patients with suspected leukocyte adhesion deficiency underwent analysis of CD18/CD11a, and CD15 expression on neutrophils to investigate defects in leukocyte adhesion molecules.

##### 2.2.3.2 Intracellular protein expression

Intracellular protein expression was assessed by flow cytometry using permeabilized lymphocytes or monocytes, with the selection of target proteins guided by the suspected underlying IEI. LRBA protein expression was evaluated in patients with clinical features suggestive of LRBA deficiency. DOCK8 protein expression was assessed in two patients with clinically suspected combined immunodeficiency and subsequently compared with the final molecular diagnosis.

#### 2.2.4 Functional assays

##### 2.2.4.1 Dihydrorhodamine 123 assay

The dihydrorhodamine 123 (DHR 123) flow cytometric assay was performed to assess neutrophil oxidative burst activity in patients with suspected chronic granulomatous disease (CGD). Peripheral blood neutrophils were stimulated with phorbol 12-myristate 13-acetate (PMA) in the presence of DHR 123 for approximately 20 minutes. Oxidative burst activity was quantified by calculating the stimulation index, defined as the ratio of fluorescence intensity in stimulated neutrophils to that in unstimulated controls. In our laboratory, a stimulation index ≥100 was considered the reference cutoff for a normal oxidative burst response.

#### 2.2.5 Diagnostic contribution of flow cytometry

The diagnostic contribution of targeted flow cytometry was retrospectively classified into three categories: strong evidence, probable evidence, and no evidence. The grading framework was developed for the present analysis based on established disease-specific immunophenotypic and functional associations reported in the literature (8, 9). Because all patients had a genetically confirmed diagnosis, the recorded flow cytometric findings were evaluated in relation to the final molecular diagnosis. Classification was performed by consensus among the study immunologists using the same grading principles across all IUIS disease groups.

Strong evidence was assigned when the flow cytometric findings demonstrated a highly characteristic immunophenotypic or functional abnormality that strongly supported the subsequently confirmed diagnostic category. Examples included characteristic T−B−NK immunophenotypes in severe combined immunodeficiency, absence or marked reduction of circulating B cells in agammaglobulinemia, increased TCRαβ double-negative T cells in autoimmune lymphoproliferative syndrome, an abnormal DHR-123 oxidative burst in chronic granulomatous disease, and markedly reduced or absent LRBA, FOXP3, DOCK8, or HLA-DR expression in the corresponding disorders.

Probable evidence was assigned when flow cytometric abnormalities were consistent with and supportive of the genetically confirmed diagnostic category but lacked sufficient specificity to strongly indicate that category. Examples included T-, B-, or NK-cell lymphopenia, reduced recent thymic emigrants, or altered B-cell subset distributions that narrowed the differential diagnosis but required integration with clinical and molecular findings.

No evidence was assigned when flow cytometric findings were normal or when the observed abnormalities did not provide meaningful support for the genetically confirmed diagnostic category.

For analyses of diagnostic contribution, flow cytometry was considered contributory when either strong or probable evidence was present. This retrospective grading was performed for descriptive and analytical purposes and did not influence the original diagnostic process, patient management, or molecular testing.

### 2.3 Genetic analyses

Genetic diagnoses were established between 2005 and 2025 at multiple clinical genetics centers using the diagnostic modalities available during the study period. Depending on the clinical indication and the period of evaluation, genetic analyses included chromosomal microarray analysis for copy number variant detection, Sanger sequencing for single-gene testing, targeted next-generation sequencing gene panels, whole-exome sequencing, and whole-genome sequencing.

### 2.4 Statistical analysis

Statistical analyses were performed using IBM SPSS Statistics version 26.0 (IBM Corp., Armonk, NY, USA). Continuous variables are presented as medians with ranges (minimum–maximum), and categorical variables as frequencies and percentages. The distribution of continuous variables was assessed using the Shapiro–Wilk test.

The association between IUIS disease categories and the flow cytometric contribution categories was evaluated using Pearson’s χ² test. As 7 of 21 cells (33.3%) had expected counts <5 (minimum expected count: 1.25), statistical significance was confirmed using the Fisher–Freeman–Halton exact test rather than relying solely on the asymptotic χ² approximation. The strength of association was quantified using Cramér’s V.

Pairwise comparisons of diagnostic contribution, defined as the proportion of patients in whom flow cytometry was retrospectively classified as providing either strong or probable evidence, were performed using Fisher’s exact test. To account for multiple testing, the resulting 21 p-values were adjusted using the Holm–Bonferroni method. All tests were two-sided, and an adjusted *p* value <0.05 was considered statistically significant.

## 3 Results

### 3.1 Baseline demographics and clinical characteristics

A total of 243 patients with genetically confirmed inborn errors of immunity (IEIs) who were evaluated between 2005 and 2025 were included in the study. Of these, 147 (60.5%) were male and 96 (39.5%) were female. The median age at symptom onset was 12 months (range, 0–168 months), the median age at initial presentation and flow cytometric evaluation was 44 months (range, 1–204 months), and the median age at molecular diagnosis was 70 months (range, 1–208 months). Parental consanguinity was documented in 99 families (40.7%), and a positive family history of IEIs was identified in 84 patients (34.6%).

Sinopulmonary infections were the most common presenting manifestation, accounting for 39.5% (n = 96) of cases, followed by invasive infections (12.8%, n = 31) and dermatological manifestations (11.9%, n = 29).

The distribution of patients according to the 2025 International Union of Immunological Societies (IUIS) classification is summarized in **Figure 1**, with predominantly antibody deficiencies representing the largest disease category (31.7%), followed by syndromic combined immunodeficiencies (27.1%) and combined immunodeficiencies (17.3%).

**Figure 1:**
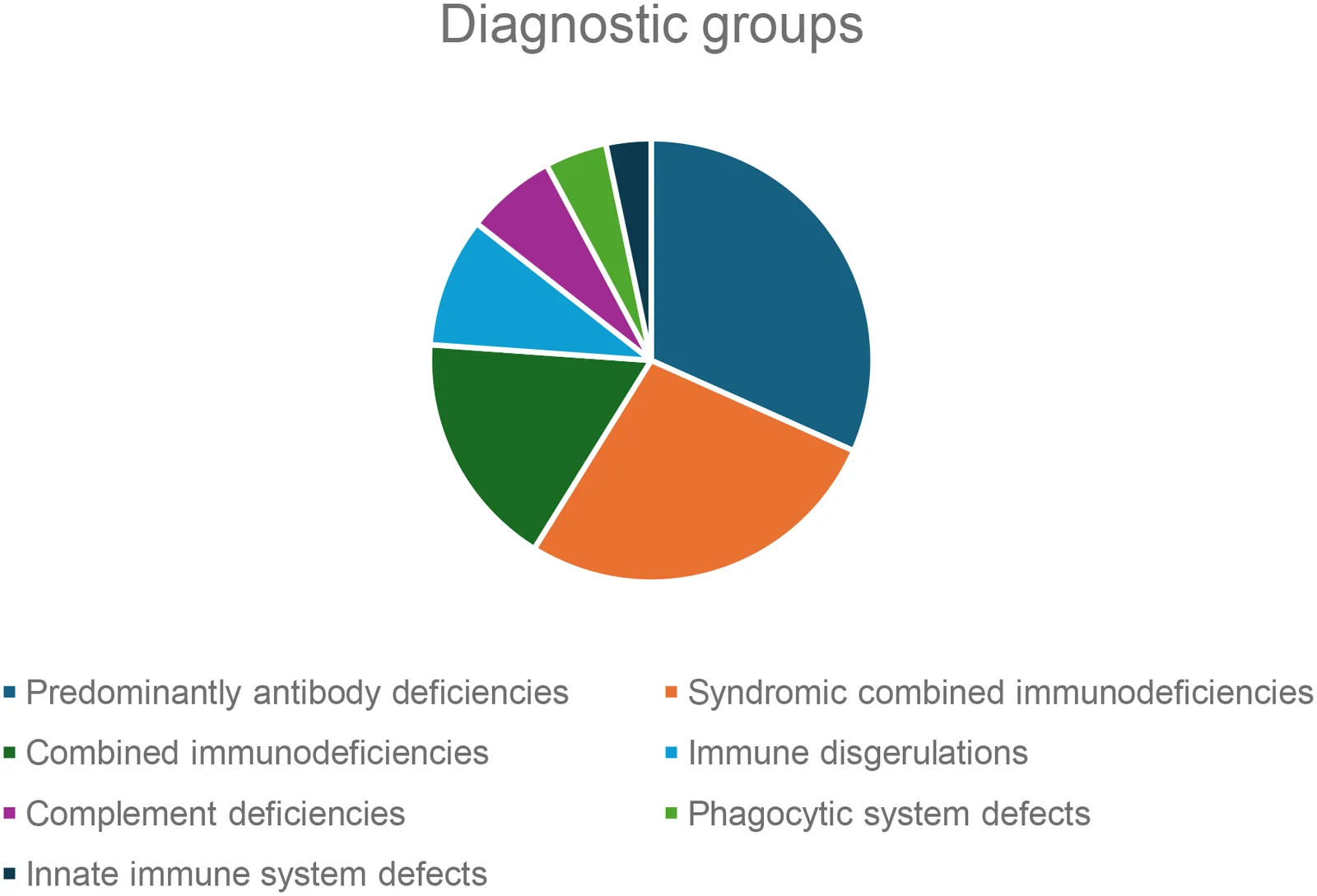
Pie chart showing proportions of diagnostic groups for immunodeficiencies, with each segment representing: predominantly antibody deficiencies, combined immunodeficiencies, complement deficiencies, innate immune system defects, syndromic combined immunodeficiencies, immune disregulations, and phagocytic system defects.

### 3.2 Diagnostic contribution of targeted flow cytometry

Targeted flow cytometry contributed to the diagnostic evaluation in 153 of 243 patients (63.0%). Among these, flow cytometric findings correctly predicted the underlying genetic diagnostic category in 41 patients (27.0%), demonstrating its value as an adjunct to molecular diagnosis.

The median age at flow cytometric evaluation was 44 months (range, 1–204 months), whereas the median age at definitive genetic diagnosis was 70 months (range, 1–208 months). In all patients, targeted flow cytometric evaluation preceded molecular confirmation. The median interval between flow cytometric evaluation and genetic confirmation was 12 months (range, 1–180 months), indicating that targeted flow cytometry frequently provided clinically informative findings before molecular confirmation.

The diagnostic performance of targeted flow cytometry across the different IUIS disease categories is summarized in **Table 2**. Diagnostic yield differed significantly among disease categories. Patients with combined immunodeficiency demonstrated a significantly higher diagnostic contribution than those with syndromic combined immunodeficiency, predominantly antibody deficiencies, and complement deficiencies (adjusted *p* < 0.05). Likewise, diseases of immune dysregulation showed a significantly higher diagnostic contribution than syndromic combined immunodeficiency and complement deficiencies, while syndromic combined immunodeficiency demonstrated a higher diagnostic yield than predominantly antibody deficiencies, defects in intrinsic and innate immunity, and complement deficiencies (all adjusted *p* < 0.05).

**Table 2:** Diagnostic contribution of flow cytometry across disease categories.

Usefulness of FCM	CID n (%)	Syndromic CID n (%)	Primary antibody deficiencies n (%)	Immune dysregulation n (%)	Phagocytic system defects n (%)	Innate immune deficiencies n (%)	Complement deficiencies n (%)
Strong evidence	15 (35.7)	–	12 (15.6)	10 (43.5)	4 (36.4)	–	–
Probable evidence	27 (64.3)	47 (71.2)	23 (29.9)	9 (39.1)	5 (45.5)	1 (12.5)	–
No evidence	–	19 (28.8)	42 (54.5)	4 (17.4)	2 (18.1)	7 (87.5)	16 (100)

FCM, flow cytometry; CID, combined immunodeficiencies. Strong evidence: a highly characteristic immunophenotypic or functional abnormality strongly supporting the genetically confirmed diagnostic category. Probable evidence: abnormalities consistent with and supportive of the confirmed category but lacking sufficient specificity to strongly indicate it. No evidence: normal findings or abnormalities providing no meaningful support for the confirmed category. Categories were assigned retrospectively using the grading criteria described in Section 2.2.5.

#### 3.2.1 Combined immunodeficiency

Targeted flow cytometry provided diagnostic information in all patients with combined immunodeficiency (42/42, 100%). Among these, 19 patients were diagnosed with severe combined immunodeficiency (SCID).

Characteristic lymphocyte immunophenotypes accurately predicted the underlying molecular defect in most SCID cases. A T−B−NK+ phenotype was identified in 10 patients and was associated with *RAG1* mutations (n = 7), *RAG2* mutation (n = 1), and *LIG4* mutations (n = 2). Four patients demonstrated a T−B−NK− phenotype, all of whom harbored *ADA* mutations. A T−B+NK− phenotype was observed in three patients and was consistently associated with *JAK3* mutations, whereas two patients with a T−B+NK+ phenotype were subsequently found to carry *CORO1A* mutations.

Beyond lymphocyte subset analysis, targeted protein expression assays further refined the diagnostic evaluation. Reduced HLA-DR expression identified three patients with major histocompatibility complex class II deficiency, in whom pathogenic variants in *CIITA, RFX5*, and *RFXAP* were subsequently confirmed. Two of the 17 patients with *DOCK8* deficiency who had numerical lymphocyte-subset abnormalities also demonstrated reduced intracellular DOCK8 protein expression. Among the remaining patients with combined immunodeficiency in whom flow cytometry supported the diagnosis, pathogenic variants were identified in *DOCK2* (n = 2) and *MSN* (n = 1). The complete distribution of genetic defects is presented in **Table 3**.

**Table 3:** Flow cytometry and genetic analysis findings in patients with IEIs.

Diagnoses	Flow cytometry data	Genetic data (n)
Numerical abnormalities of lymphocyte subsets		
Agammaglobulinemia — X-linked	B cell absence	*BTK* (12)
Agammaglobulinemia — autosomal recessive	B cell absence	*IGLL1* (1)
SCID/CID	T, B, NK cell deficiencies	*DOCK2* (2), *DOCK8* (17)¹, *MSN* (1), *LIG4* (2), *RAG1* (7), *CORO1A* (2), *RAG2* (1), *JAK3* (3), *ADA* (4)
ALPS	Increase in TCRαβ CD3+CD4−CD8− T cells	*CASP10* (1), *FASLG* (1), *FAS* (2)
Syndromic CID	Low thymic emigrants	22q11.2 deletion (13)
Syndromic CID	T, B cell deficiencies	*ATM* (20), *NBN* (2), *BLM* (2), *STAT3* (1), *STAT5B* (1), *TCN2* (3), *WAS* (3), *PNP* (2)
Primary Antibody Deficiencies	Changes in B cell and B cell subsets	*TNFRSF13B* (15), *TNFRSF13C* (2), *TNFSF13* (2), *NFKB2* (1), *PIK3R1* (1), *AICDA* (1)
Phagocytic system defects	T, B, NK cell deficiencies	*WDR1* (3), *VPS45* (1), *GFI1* (1)
Immune dysregulation	T, B, NK cell deficiencies	*BACH2* (1), *AP3B1* (1), *TNFRSF9* (2), *CTLA4* (2), *LRBA* (1)², *STAT3* (3)
Innate immune deficiencies	T, B, NK cell deficiencies	*IL12RB1* (1)
Specific cell surface protein		
CID	Low HLA-DR expression + low CD4	*RFX5* (1), *CIITA* (1), *RFXAP* (1)
Intracellular specific protein		
IEI with immune dysregulation	LRBA protein expression defect	*LRBA* (3)²
IEI with immune dysregulation	FOXP3 protein expression defect	*FOXP3* (2)
CID	DOCK8 expression defect	*DOCK8* (2)¹
Functional abnormalities		
Phagocytic system defects (Chronic Granulomatous Disease)	Abnormal DHR test	*CYBA* (2), *CYBB* (2)

¹ Two of the 17 patients with DOCK8 deficiency who had numerical lymphocyte-subset abnormalities also demonstrated reduced intracellular DOCK8 protein expression. These patients are represented in both rows but are counted only once in the total number of patients with DOCK8 deficiency (n = 17). ² The LRBA entries in the numerical-abnormalities row (n = 1) and the intracellular-protein row (n = 3) represent four distinct patients with no overlap: one identified through lymphocyte-subset abnormalities and three through reduced intracellular LRBA protein expression. ALPS, Autoimmune lymphoproliferative syndrome; CID, combined immunodeficiency; DHR: dihydrorhodamine; SCID, severe combined immunodeficiency.

#### 3.2.2 CIDs associated with syndromic features

Targeted flow cytometry contributed to the diagnostic evaluation in 47 of 66 patients (71.2%) with combined immunodeficiencies associated with syndromic features. The most frequent abnormalities included T-cell lymphopenia, B-cell lymphopenia, and reduced recent thymic emigrants, all of which supported the underlying molecular diagnosis. The corresponding genetic defects are summarized in **Table 3**.

#### 3.2.3 Predominantly antibody deficiencies

Among patients with predominantly antibody deficiencies, targeted flow cytometry contributed to the diagnostic evaluation in 35 of 77 patients (45.5%) (**Table 2**). Agammaglobulinemia was identified by the absence or profound reduction of circulating B cells in 12 patients (15.6%), while abnormalities in peripheral B-cell subsets supported the diagnosis in an additional 23 patients (29.9%).

#### 3.2.4 Diseases of immune dysregulation

Targeted flow cytometry contributed to the diagnostic evaluation in 19 of 23 patients (82.6%) with diseases of immune dysregulation and provided a definitive diagnosis in 10 patients (43.5%).

Reduced intracellular LRBA protein expression identified three patients with genetically confirmed *LRBA* deficiency, while absent or markedly reduced FOXP3 expression established the diagnosis of IPEX syndrome in two patients. In an additional seven patients, abnormalities involving T-, B-, and NK-cell compartments supported the diagnosis of immune dysregulation disorders.

Autoimmune lymphoproliferative syndrome (ALPS) was identified in four patients based on an increased proportion of TCRαβ double-negative T cells. Subsequent molecular analysis demonstrated *CASP10* (n = 1), *FASLG* (n = 1), and *FAS* (n = 2) mutations.

#### 3.2.5 Congenital defects of phagocyte number or function

Targeted flow cytometry contributed to the diagnostic evaluation in 9 of 11 patients (81.8%) with phagocytic defects and provided a definitive diagnosis in four patients (36.4%). Neutrophil oxidative burst assessment using the DHR 123 assay represented the principal diagnostic tool in this group, while abnormalities in T-, B-, and NK-cell subsets provided additional supportive findings. In contrast, flow cytometry did not identify diagnostic abnormalities in a patient carrying the *ELANE* mutation and one patient with *HAX1* mutations.

Beyond assessment of the neutrophil oxidative burst, T-, B-, and NK-cell subset abnormalities provided non-specific but supportive information in patients with congenital defects of phagocyte number or function. Such abnormalities may indicate broader immune involvement rather than an isolated neutrophil defect, particularly in disorders caused by genes with functions extending across multiple hematopoietic lineages. In our cohort, this was relevant to defects involving *GFI1*, *VPS45*, and *WDR1*, which have roles in hematopoietic development, intracellular trafficking, or cytoskeletal regulation beyond the neutrophil compartment. This was particularly evident in patients with *WDR1* deficiency, in whom marked B-cell lymphopenia provided evidence of immune involvement extending beyond the phagocytic compartment. Concomitant T-, B-, or NK-cell abnormalities therefore provided additional phenotypic context and could help distinguish broader or syndromic immune defects from disorders predominantly affecting neutrophils. These findings were not disease-specific and should not be interpreted as diagnostic in isolation; rather, lymphocyte subset analysis served as a complementary component of immunophenotyping alongside neutrophil-specific functional assessment and molecular testing.

#### 3.2.6 Defects in intrinsic and innate immunity

Among patients with defects in intrinsic and innate immunity, targeted flow cytometry contributed to the diagnosis in one patient with an *IL12RB1* mutation by demonstrating T-cell deficiency. No diagnostically relevant flow cytometric abnormalities were detected in the remaining seven patients (87.5%).

#### 3.2.7 Complement deficiencies

Targeted flow cytometry did not provide diagnostically relevant findings in any patient with complement deficiency (0/16).

## 4 Discussion

The diagnosis of inborn errors of immunity (IEIs) remains challenging because of their marked clinical and immunological heterogeneity. In this 20-year single-center cohort of genetically confirmed IEIs, we found that targeted flow cytometry contributed to the diagnostic evaluation in 63% of patients and correctly predicted the underlying molecular disease category in 27% of cases with abnormal flow cytometric findings. Notably, flow cytometric abnormalities were identified a median of 12 months before genetic confirmation, underscoring the ability of targeted immunophenotypic and functional assays to provide clinically actionable information early in the diagnostic pathway. These findings are consistent with previous reports highlighting the value of flow cytometry as an indispensable component of the diagnostic work-up for IEIs and further support its role as a bridge between clinical suspicion and definitive molecular diagnosis (11).

Delayed molecular diagnosis remains one of the major challenges in the management of IEIs, particularly in healthcare settings where access to comprehensive genetic testing may be limited or prolonged. Similar diagnostic delays have been reported by the Middle East and North Africa (MENA) Registry, which documented a mean age at disease onset of 36 months and an average diagnostic delay of 41 months (12). In our cohort, targeted flow cytometry provided diagnostically informative findings a median of 12 months before molecular confirmation, highlighting its potential to bridge this critical interval. By identifying disease-specific immunophenotypic and functional abnormalities early in the diagnostic process, targeted flow cytometry facilitates timely clinical decision-making and supports the initiation of appropriate interventions, including antimicrobial prophylaxis, immunoglobulin replacement therapy, and hematopoietic stem cell transplantation, before definitive molecular results become available. The clinical value of this lead time is greatest in the disorders in which flow cytometry is itself essentially diagnostic and in which delay carries the highest risk, and in our cohort it consistently translated into earlier management. In all 19 patients with severe combined immunodeficiency, a characteristic T−B−NK± immunophenotype prompted immediate protective measures — reverse isolation, avoidance of live vaccines and non-irradiated blood products, immunoglobulin replacement, and antimicrobial prophylaxis — together with urgent referral for hematopoietic stem cell transplantation, all initiated on the basis of the immunophenotype before molecular confirmation. In all four patients with chronic granulomatous disease, an abnormal DHR 123 assay functionally established the diagnosis and prompted antibacterial and antifungal prophylaxis without awaiting the causative gene. In all 13 patients with agammaglobulinemia, the absence of circulating B cells together with hypogammaglobulinemia supported the initiation of immunoglobulin replacement before genetic confirmation. Beyond these groups, flow cytometric evidence of immune dysregulation — including reduced LRBA or FOXP3 expression and abnormal lymphocyte compartments — similarly guided the early introduction of directed treatment, such as immunosuppressive and targeted therapy, in advance of molecular results. In each of these scenarios molecular analysis subsequently confirmed the specific defect but was not a prerequisite for beginning appropriate treatment. Given the median 12-month interval between flow cytometric and molecular diagnosis, targeted flow cytometry meaningfully advanced these management decisions and, in the most time-critical disorders, allowed potentially life-saving interventions to begin months earlier than genetic testing alone would have permitted.

The broad diagnostic utility of flow cytometry in IEIs extends beyond the identification of quantitative lymphocyte abnormalities. By combining detailed immunophenotypic analysis with the assessment of cell surface, intracellular, and nuclear protein expression, as well as functional immune assays, flow cytometry provides disease-specific information that complements conventional laboratory investigations and molecular testing. These capabilities not only facilitate the recognition of characteristic immunological signatures but also improve disease classification and guide the selection of targeted genetic analyses, particularly in clinically heterogeneous IEIs (8, 13).

The distribution of IEI categories in our cohort was broadly consistent with previous regional reports. Predominantly antibody deficiencies represented the largest disease group (31.7%), similar to the findings of the Middle East and North Africa (MENA) Registry, in which they accounted for 41.2% of all reported IEIs (12). However, in a study by Belaid et al. evaluating a cohort of 807 adult and pediatric patients, combined immunodeficiencies were reported as the most prevalent group, accounting for 33.8% of cases (14). The parental consanguinity rate of 40% observed in our cohort further reflects the high burden of autosomal recessive IEIs reported in populations with frequent consanguineous marriages (15). The relatively high rate of consanguineous marriage in our study group (40.7%) necessitates caution in generalizing these findings to populations where consanguineous marriage is uncommon. In populations with lower consanguinity rates, the case mix may include a relatively greater proportion of predominantly antibody deficiencies, including common variable immunodeficiency, for which flow cytometry is often supportive rather than predictive of a specific molecular diagnosis. Nevertheless, the biological basis of disease-specific flow cytometric assays is not expected to be inherently altered by background consanguinity. For example, a Korean single-center cohort with a low consanguinity rate showed a similar category-bound pattern with a stronger diagnostic contribution in severe cellular and phagocytic defects and a predominantly supportive role in humoral disorders (16). Therefore, while population structure may affect the distribution of IEIs and thus the overall diagnostic yield of flow cytometry, its targeted disease-specific utility is likely to remain clinically informative in different populations.

The diagnostic performance of targeted flow cytometry differed markedly across IUIS disease categories, reflecting the underlying immunopathology of individual IEIs. In our cohort, flow cytometry contributed to the diagnostic evaluation of all patients with combined immunodeficiency, 82.6% of those with diseases of immune dysregulation, and 81.8% of patients with phagocytic defects, whereas its contribution was more limited in predominantly antibody deficiencies (45.5%). These findings are consistent with previous studies demonstrating that the clinical value of flow cytometry is greatest in disorders characterized by distinct cellular or functional immunological abnormalities. In a similar manner, a Korean tertiary care center stratified flow cytometric findings into “strong diagnostic evidence” for severe cellular and phagocytic defects—such as severe combined immunodeficiency, X-linked agammaglobulinemia, and leukocyte adhesion deficiency—while considering its role mainly supportive or monitoring-oriented in more heterogeneous humoral disorders, including common variable immunodeficiency (16). Similarly, an Algerian cohort of 670 patients reported that flow cytometry enabled meaningful diagnostic classification in 76.7% of cases by identifying characteristic structural and functional immune abnormalities (15). Collectively, these observations support the concept that the diagnostic yield of targeted flow cytometry depends largely on the biological characteristics of each IEI category rather than the technique itself.

Although targeted flow cytometry did not contribute to the diagnosis of complement deficiencies in our cohort, this finding should be interpreted in the context of the flow cytometric assays available during the study period. In contrast, the Algerian cohort demonstrated that targeted assessment of complement regulatory proteins, including CD46 and CD55 expression, can provide valuable diagnostic information in selected disorders such as atypical hemolytic uremic syndrome and protein-losing enteropathy (15). Conventional lymphocyte subset analysis and the DHR-123 test do not evaluate the complement system, therefore, targeted flow cytometry did not provide a diagnostic contribution in the complement deficiency group. However, specialized flow cytometric assessment of complement-related cell-surface proteins may provide diagnostically relevant information in selected complement regulatory defects, and most isolated complement deficiencies are diagnosed with quantitative and functional complement assays. Tests to evaluate complement-related surface proteins were not routinely available during the study period and were not represented in our panel. The lack of contribution in this category reflects the limitations of the specific panel evaluated rather than a universal limitation of flow cytometry. More broadly, the diagnostic contribution of conventional lymphocyte subset analysis and constitutive protein-expression assays is expected to be limited in disorders in which baseline cellular composition and protein expression are preserved. These may include predominantly antibody deficiencies with preserved B-cell numbers, selected defects involving cytokine-signaling or innate immune pathways, and many autoinflammatory disorders. In such conditions, pathway-specific functional assays, biochemical or complement studies, and molecular testing may provide greater diagnostic information. Importantly, some functional immune defects can be investigated using specialized stimulation-based flow cytometric assays; therefore, limited yield from routine immunophenotyping should not be interpreted as a limitation of all flow cytometric approaches.

Our findings are consistent with those of several large international cohorts, supporting the role of targeted flow cytometry as a central component of the diagnostic evaluation of IEIs. In the present study, flow cytometry contributed to the diagnosis in 153 of 243 genetically confirmed patients (63%) and correctly predicted the underlying molecular disease category in 41 patients (27%). Comparable diagnostic performance has been reported in other large cohorts. An Algerian study of 670 patients demonstrated that flow cytometry contributed to diagnosis and disease classification in 76.7% of cases, including 104 patients in whom a definitive diagnosis was established by flow cytometric analysis alone (15). Similarly, an Egyptian cohort of 1,019 patients reported diagnostic and classification rates of 73% and 28%, respectively (10). In a Korean tertiary referral center, abnormal flow cytometric findings prompted the diagnosis of IEIs in a substantial proportion of patients and were sufficient to establish the diagnosis before molecular testing in selected cases (16). Taken together, these observations demonstrate that the diagnostic value of targeted flow cytometry is reproducible across geographically and ethnically diverse IEI populations despite differences in disease distribution and laboratory practice.

These results should not be interpreted as suggesting that flow cytometry can replace molecular testing. In our study group, flow cytometry alone correctly predicted the confirmed genetic category in 41 patients (27% of those with contributing findings and 17% of the entire group); this rate corresponds to cases providing strong diagnostic evidence in **Table 2**. Therefore, in most patients, its role was to support and guide the diagnostic process rather than directly determining the category. Even if the immunophenotype does not fit a single genetic category, an abnormal result can narrow the differential diagnosis, indicate which functional pathway is affected, and help prioritize and sequence subsequent genetic investigations. We believe that its consideration would be particularly beneficial in centers where targeted panels and exome sequencing are limited in access and cost. One of the strengths of targeted flow cytometry is its ability to provide functional evidence (including interpretation of variants of uncertain significance) that sequencing cannot, in disorders with distinct cellular or functional signatures such as severe combined immunodeficiency, chronic granulomatous disease, LRBA deficiency, and IPEX syndrome. Therefore, we view targeted flow cytometry not as an alternative, but as a supportive, guiding, and sometimes decisive component of a diagnostic algorithm that results in molecular validation.

Although molecular genetic testing remains the reference standard for confirming IEIs, our findings illustrate that targeted flow cytometry provides clinically relevant information considerably earlier in the diagnostic pathway. Compared with next-generation sequencing–based approaches, flow cytometry offers a rapid, functional, and relatively cost-effective method for evaluating immune defects, particularly when immediate therapeutic decisions are required (9, 15). Furthermore, while next-generation sequencing, whole-exome sequencing, and whole-genome sequencing have transformed the molecular diagnosis of IEIs, these approaches remain limited by cost, turnaround time, and the interpretation of complex or uncertain genetic variants (17, 18). Functional immunological assessment by flow cytometry therefore complements molecular testing by providing direct evidence of immune dysfunction and by helping to prioritize candidate genes and guide subsequent genetic investigations.

Functional flow cytometric assays provide direct evidence of immune cell dysfunction that cannot be inferred from genetic data alone. Representative examples include the assessment of neutrophil oxidative burst using the dihydrorhodamine (DHR) assay and the evaluation of natural killer cell cytotoxicity through degranulation assays (16). These complementary functional data are particularly valuable for the interpretation of variants of uncertain significance identified by broad genomic sequencing approaches, as they help establish the biological relevance of candidate variants and strengthen genotype–phenotype correlations (17–19).

Although targeted flow cytometry provides substantial diagnostic value in IEIs, its limitations should also be recognized. One of the key constraints of flow cytometry is that the detection of a protein at the cellular level does not necessarily reflect its functional integrity, as certain missense variants may produce structurally preserved but biologically inactive proteins (15). In addition, the technique depends on high-quality fresh specimens, standardized pre-analytical processing, and expert interpretation using appropriate age-matched reference ranges. Furthermore, because targeted antibody panels are generally selected according to the initial clinical phenotype, atypical presentations or unexpected molecular defects may remain undetected if complementary investigations are not performed (16). These limitations reinforce the importance of integrating flow cytometry with comprehensive clinical evaluation and molecular genetic testing rather than considering either approach in isolation.

Molecular genetic testing also has important limitations that should be considered when establishing the diagnosis of IEIs. Depending on the analytical platform used, pathogenic variants such as large genomic deletions, complex structural rearrangements, or variants located within pseudogene regions may be missed or remain difficult to interpret (9, 17). In addition, relatively long turnaround times may delay therapeutic decision-making, particularly in critically ill patients requiring urgent intervention. These limitations further emphasize the complementary roles of molecular genetics and targeted flow cytometry, with functional immunological assessment providing clinically actionable information while definitive molecular confirmation is pending.

This study has several limitations that should be acknowledged. The range of targeted flow cytometric assays available during the study period was determined by local laboratory capacity and did not encompass all potentially informative disease-specific assays. Moreover, the grading of flow cytometric contribution was performed retrospectively with knowledge of the final molecular diagnosis rather than through a prospectively predefined, blinded assessment. Although the categories were based on established disease-specific immunophenotypic and functional associations and were applied by consensus using consistent grading principles, knowledge of the molecular diagnosis may have influenced the interpretation of flow cytometric findings and may therefore have increased the estimated diagnostic contribution. Consequently, sensitivity, specificity, and positive and negative predictive values cannot be calculated, and the reported contribution rates should be interpreted as descriptive measures of the relationship between flow cytometric findings and the final molecular diagnosis rather than as estimates of diagnostic performance. Nevertheless, the large cohort of genetically confirmed IEIs and the long-term experience with targeted flow cytometric evaluation represent important strengths of the present study.

Our findings also suggest several directions for future research. Expanding the availability of disease-specific flow cytometric assays, particularly those targeting complement regulatory proteins and additional functional immune pathways, may further improve the diagnostic performance of targeted flow cytometry. Furthermore, continued integration of high-dimensional flow cytometry with molecular sequencing and other functional immunological approaches is likely to refine genotype–phenotype correlations and facilitate more precise characterization of IEIs. As novel biomarkers continue to emerge, targeted flow cytometry is expected to play an increasingly important role not only in diagnosis but also in disease classification and precision medicine for patients with IEIs.

## 5 Conclusion

In conclusion, this 20-year single-center study demonstrates that targeted flow cytometry is an indispensable component of the diagnostic evaluation of inborn errors of immunity. By integrating immunophenotyping, protein expression analysis, and functional immune assays, targeted flow cytometry provides rapid, clinically actionable information that complements molecular genetic testing and facilitates earlier disease recognition and management. Although genetic testing remains the reference standard for definitive molecular diagnosis, our findings show that targeted flow cytometry substantially improves diagnostic precision by guiding disease classification, prioritizing genetic investigations, and supporting timely therapeutic decision-making. Together, these findings reinforce the value of integrating targeted flow cytometry into routine diagnostic algorithms for patients with suspected IEIs.

## Data Availability

The raw data supporting the conclusions of this article will be made available by the authors, without undue reservation.
